# Is Regular Radiographic Upper Urinary Tract Imaging for Surveillance of Non-Muscle Invasive Bladder Cancer Justified?

**DOI:** 10.3390/cancers14225586

**Published:** 2022-11-14

**Authors:** Uwe Bieri, Benedikt Kranzbühler, Burkhardt Seifert, Birgit Maria Helmchen, Alexander Gu, Basil Kaufmann, Dejan Lavrek, Thomas Scherer, Marian S. Wettstein, Cédric Poyet, Thomas Hermanns

**Affiliations:** 1 Department of Urology, University Hospital Zürich, University of Zürich, 8091 Zürich, Switzerland; 2 Epidemiology, Biostatistics and Prevention Institute, University of Zürich, 8006 Zürich, Switzerland; 3 Department of Pathology, and Molecular Pathology, University Hospital of Zurich, University of Zurich, 8091 Zürich, Switzerland

**Keywords:** urologic neoplasms, upper-urinary-tract urothelial cancer, surveillance, diagnostic imaging

## Abstract

**Simple Summary:**

Patients with non-muscle invasive (NMI) urothelial bladder cancer (BC) are more likely to have a second cancer in the upper urinary tract (UTUC). During bladder cancer follow-up, UTUC can be detected early by regular imaging of the upper urinary tract. However, there is little evidence that this strategy is useful. We performed a retrospective analysis of patients with NMIBC treated at our center between 2003 and 2016. Our results show that regular imaging has a low detection rate and that UTUCs were only found in individuals who already had a high-risk disease, suggesting that only these individuals may require upper-tract surveillance. In the future, risk-adjusted follow-up strategies should be explored.

**Abstract:**

Patients with non-muscle invasive (NMI) urothelial bladder cancer (BC) are at increased risk for the development of a secondary upper-urinary-tract urothelial carcinoma (UTUC). We aimed to assess the usefulness of routine upper-tract imaging surveillance during NMIBC follow-up in a patient cohort of a tertiary academic center. All routine upper-tract-imaging scans using computerized tomography urography (CTU) between 2003 and 2016 were assessed for UTUC detection. A total of 315 patients were analyzed. Initial tumor stage was Ta in 207 patients (65.7%), T1 in 98 patients (31.1%) and pure CIS in 10 patients (3.2%). A total of 149 (47.3%) presented with low-grade (LG), and 166 (52.7%) with high-grade (HG) disease. Median follow-up was 48 months (IQR: 55). Four patients (1.2%) were diagnosed with UTUC during follow-up. All four patients presented with initial Ta HG BC. Two of the patients (50%) were diagnosed by routine upper tract imaging. The other two patients were diagnosed after development of symptoms. The 5- and 10-year UTUC-free survival was 98.5% (standard error (SE) 0.9) and 97.6% (SE 1.3), respectively. UTUCs were detected exclusively in patients with initial HG disease, indicating that upper-tract surveillance might only be necessary in these patients.

## 1. Introduction

Urothelial bladder cancer (BC) is the tenth most prevalent type of cancer overall [1]. Current diagnostic tools to detect bladder cancer are cystoscopy and cytology. At the time of diagnosis, the majority of all detected BCs are non-muscle-invasive (NMI) [2]. Ultimately, 20% of NMIBC will develop into invasive cancers, and 50% of those who already have invasive disease will develop metastases. Metastatic BC exhibits a high mortality rate despite systemic therapy, with a median survival of 12–15 months and a 5% 5-year survival rate [3]. The current gold standard of care for NMIBC is transurethral resection of bladder tumor (TURBT) with or without intravesical therapy, such as mitomycin C or Bacillus Calmette–Guerin [2]. Bladder cancer is the most costly cancer to treat from diagnosis to death due to the high rates of progression and recurrence and the necessity for an intensive follow-up schedule of the bladder and the upper urinary tract [4]. Patients diagnosed with BC are also at increased risk to develop upper urinary tract urothelial carcinoma (UTUC) [5]. Patients considered to have an increased risk for the development of UTUC are those within a higher risk group according to bladder tumor risk group classification based on the European Organization for Research and Treatment of Cancer (EORTC) tables [6]. Depending on risk-group assignment, the risk of developing a secondary UTUC has been reported to be between 0.7% and 25% [7,8,9,10].

International guidelines recommend regular upper urinary tract imaging in yearly or bi-yearly intervals in patients being followed for intermediate- to high-risk BC [11]. Computerized tomography urography (CTU) was demonstrated to have the highest diagnostic accuracy for UTUC detection and has replaced excretory urography as the imaging modality of choice [12,13]. Magnetic resonance urography (MRU) can serve as an alternative in patients who are not suitable for CTU [14].

The recommendations for regular upper-tract imaging are based on a limited number of retrospective studies [2]. Therefore, the shortcomings of regular imaging must be considered. Periodically performed imaging in the context of the relatively low incidence of UTUC [7,8,9,10] may unnecessarily expose patients to the potential side effects of radiation [15]. Furthermore, incidental findings of CTUs, which require further work-up, increase health-care costs and put patients at risk of overtreatment [16].

The aim of the present investigation was to retrospectively evaluate the usefulness of routine upper-urinary-tract imaging during follow-up of NMIBC.

## 2. Patients and Methods

We performed a retrospective analysis of all patients followed for NMIBC in our tertiary care academic center between 2003 and 2016. The study protocol was approved by the local ethics committee (BASEC Nr. 2016-00158).

### 2.1. Patient Characteristics

All patients treated and followed for NMIBC at our institution or referred for follow-up of NMIBC were included in the present analysis. Recorded patient characteristics included age, gender, initial tumor stage and grade, as well as the presence of concomitant carcinoma in situ (CIS), according to the 2004 World Health Organization classification [17]. In patients diagnosed before 2004, tumor grading was based on the previous classification systems (WHO 1973) [18]. G1 was considered low-grade, and G3 was considered high-grade. All cases with G2 were reevaluated by a specialized uropathologist (BH) and reclassified according to the 2004 system. Additionally, overall follow-up time from diagnosis to the last available consultation or death, as well as the highest tumor stage and grade during follow-up, was documented. Exclusion criteria were previous or synchronous diagnosis of UTUC. UTUC was defined as histologically confirmed urothelial carcinoma of the ureter, the renal pelvis or the renal calyceal system.

### 2.2. Abdominal Imaging Characteristics

All performed abdominal imaging scans during the follow-up were reviewed independently of the indication for imaging. For our primary analysis, imaging was classified as routine surveillance imaging if the following criteria were met: (1) abdominal scan was performed only for upper-urinary-tract surveillance, (2) abdominal scan was performed according to a urography protocol, (3) no clinical symptoms were present at the time of imaging (e.g., micro- or macrohematuria, positive urine cytology, pain, hydronephrosis). CTU for the evaluation of the upper urinary tract was performed according to the institutional guidelines as a three-phase acquisition with a single bolus injection of contrast media and a two-phase acquisition with a single split bolus injection. According to the CTU Working Group of the European Society of Urogenital Radiology [19], our radiology department uses the three-phase CTU protocol in patients with a high risk of UTUC and the two-phase CTU protocol in patients with a low to intermediate risk of UTUC. In patients with an impaired renal function or with other contraindications for iodinated contrast, medium magnet resonance imaging (MRI) with gadolinium contrast was performed instead.

### 2.3. Statistical Analysis

Statistical analysis was performed using IBM SPSS Statistics, Version 23.0 (IBM Corp, Armonk, NY, USA) and R, Version 4.2.1 (R Foundation for Statistical Computing, Vienna, Austria). Data is shown as median (interquartile range) or number (percent). Follow-up time was calculated from the time of diagnosis of the first bladder tumor to the last available follow-up. Kaplan–Meier curves were calculated to assess 5- and 10-year UTUC-free survival.

## 3. Results

### 3.1. Patient Characteristics

Overall, 315 patients were included in this analysis. Patient characteristics are shown in Table 1. Median age of the patients was 66 years (Range: 24–98) and 253 patients (80.3%) were male. The initial tumor stage was Ta in 207 patients (65.7%), T1 in 98 patients (31.1%) and pure CIS in 10 patients (3.2%). Primary LG disease was recorded in 149 patients (47.3%), whereas primary HG disease was detected in 166 patients (52.7%). The number of patients with recurring NMIBC was 43 (13.7%). Recurrences with a secondary upgrade from LG to HG BC were seen in 15 patients (4.8%). Median follow-up was 48 months (Interquartile range: 55). However, some patients were followed for more than 60 months (32.1%), 120 months (5.4%), 180 months (1.6%) and 240 months (0.3%), respectively.

### 3.2. UTUC Detection during NMIBC Follow-Up

Overall, 396 abdominal imaging scans were performed, of which 230 were classified as routine upper-tract imaging surveillance. Only 4 patients (1.2%) developed a secondary UTUC during follow-up, with a median time to diagnosis of 37 months (Range: 8–63). Patient characteristics of these 4 patients are summarized in Table 2. Two UTUCs were detected using routine upper tract imaging. One patient was a 68-year-old female followed for initial Ta HG NMIBC without concomitant CIS. During follow-up, the patient developed multiple local recurrences. Upper-tract imaging surveillance revealed a suspicious lesion in the left ureter at the level of the bifurcation of the common iliac arteries and work-up histology confirmed a Ta, LG UTUC.

The second patient detected by routine upper urinary tract imaging surveillance was a 65-year-old male patient with an initial Ta HG NMIBC. Due to an impaired renal function, MRI was used to perform upper-tract imaging surveillance. Routine upper-urinary-tract imaging showed a concentric narrowing of the right ureter in the transition zone between the upper and middle third. These findings were not visible in the additionally performed ultrasound examination. Based on suspicious MRI findings, ureter biopsy was performed and confirmed a T1, LG UTUC. Both bladder wash cytology (BWC) and selective upper-tract (UT) cytology were negative prior to diagnosis of UTUC.

The two other patients were diagnosed using other investigations during surveillance after manifestation of symptoms (morning sickness, loss of appetite: *n* = 1, macrohematuria: *n* = 1) and subsequent work-up. The first patient, a 74-year-old male presented with hematuria in the presence of negative cystoscopy. Further diagnostic work-up with CT urography revealed a new mass in the upper caliceal group of the right kidney. This finding triggered selective right ureteral sampling and led to the detection of malignant urothelial cells. Nephroureterectomy was conducted and histology confirmed a pT1 HG cancer. The second patient, an 80-year-old female presented with morning sickness and loss of appetite. Ultrasound examination demonstrated left-sided hydronephrosis and a subsequently performed CTU showed an intraluminal mass in the respective ureter. BWC and selective cytology of the right UT showed the presence of malignant cells, consecutive biopsy confirmed the presence of a Ta LG tumor and analysis of the nephroureterectomy specimen revealed a pT3 HG UTUC.

A total of 115 routine CTUs had to be performed to detect one UTUC. Restricting the analysis to the HG BC subgroup, 77 routine CTUs had to be performed for the detection of one UTUC.

In three patients with UTUC during follow-up histological sections of the initially diagnosed NMIBC were available for review by a specialized uropathologist (BH). Interestingly, all three cases had only focal high-grade areas referring to a G2 grading of the former (1973) WHO classification. None of the cases met the full criteria for a G3 cancer.

**Figure 1:**
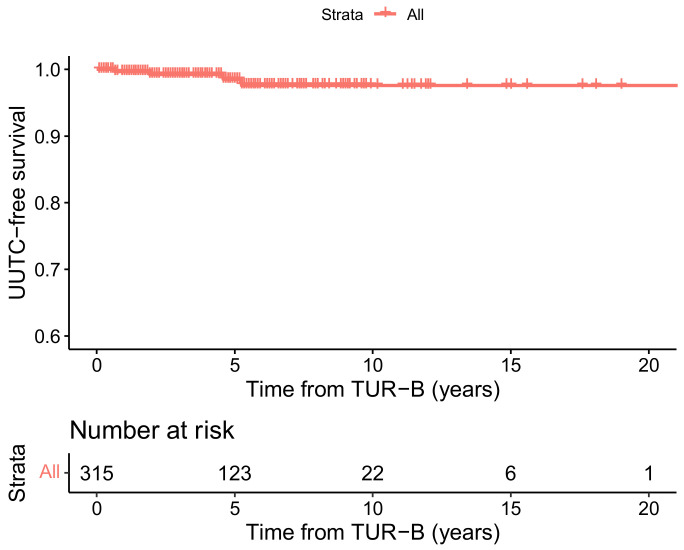
Kaplan–Meier curve for UTUC free survival. The red band bordering the curve marks the 95% confidence interval; censored subjects are indicated as tick marks.

The Kaplan–Meier curve for UTUC free survival is shown in Figure 1. The calculated overall 5- and 10-year UTUC-free survival was 98.5% (standard error (SE) 0.9) and 97.6% (SE 1.3), respectively. An additional Kaplan–Meier curve (Supplementary Figure S1), subdivided into patients with initial HG and LG NMIBC, can be found in the Supplementary Materials.

## 4. Discussion

Our analysis revealed a cumulative incidence of UTUC of only 1.2% in the entire cohort and 2.4% in the HG subgroup during surveillance of NMIBC. Only half of the patients diagnosed with UTUC during surveillance were detected by routine upper-tract imaging without any other suspicious test results (ultrasound, cytology, hematuria); the other half was diagnosed following work-up of symptoms or clinical signs.

The incidence of UTUC in patients followed for different risk categories of NMIBC is of importance to define useful upper-urinary-tract surveillance strategies. Among the 315 patients analyzed in our study, only four patients (1.2%) developed a secondary UTUC during follow-up. Golabesk et al. analyzed 704 patients with primary NMIBC and 10 years of follow-up and detected an upper-urinary-tract recurrence in 2.4% of the patients [8]. Oldbring et al. reported UTUC detection in 1.7% of 657 patients with primary bladder cancer and the same follow-up time [20]. Holmang et al. reported an UTUC detection in 2.4% of 680 patients followed for bladder cancer up to 5 years [21]. Focusing on high-risk patients only, the risk of secondary UTUC may increase up to 7.5% in a contemporary multi-center cohort [22]. In summary, studies with a more extended follow-up period report a higher incidence of UTUC, as do studies with selective high-risk populations.

Commonly reported risk factors for the development of secondary UTUC are: recurrent bladder tumors, multiple tumors at diagnosis, grade and T stage [6,23,24,25]. All UTUCs in our cohort were detected in patients with HG NMIBC, and therefore were considered high-risk. Remarkably, the pathology review of the available specimens of the bladder tumors revealed that HG patterns were only focally present. Considering that the general conformity between pathologists in the staging and grading of urothelial carcinoma is only 50–60% [26], a certain number of G2 tumors may have been incorrectly classified as LG tumors in other cohorts and, as a consequence, the risk of UTUC recurrence may have been overestimated in patients with LG disease [7,27].

Considering the time of follow-up and the discussed studies [8,9,21,27,28,29,30], no intervals of increased or decreased risk of UTUC in NMIBC follow-up can be identified over time. A circumstance that further complicates the comparability of the discussed studies is the different handling or valuation of recurrences in the bladder when establishing reference timeframes in the development of follow-up strategies, from first diagnosis of NMIBC vs. from the date of the most recent recurrence, since recurrence increases the risk of UTUC [24]. This phenomenon has not attracted considerable attention to date, but its consideration will certainly be important in the future to optimize follow-up strategies for UTUC.

Given our results, whether a risk-based approach, taking into account tumor grade, results of ultrasound, results of cytology and clinical signs, e.g., presence of hematuria prior to CTU opposed to fixed follow-up schedules, might be sufficient to detect UTUC in the follow-up of NMIBC without missing the curative intervention interval should be investigated. More selective CTU indications might reduce the negative impact of surveillance CTUs (radiation and contrast agent exposure, potentially unnecessary exams for incidental findings), particularly given the number of unnecessary surveillance CTUs that is required to identify one additional UTUC. Putting this line of reasoning in perspective; Smith-Bindman et al. postulate in their study that 1 in 700 females and 1 in 660 males at age 60 undergoing multiphase abdomen-pelvis ct-scanning are estimated to develop a radiation-induced cancer [15]. Consider our finding that a total of 115 routine CTUs had to be performed to detect one UTUC, then the detection of 6 secondary UTUCs are statistically related to one radiation-induced cancer.

Our study has limitations: Firstly, based on the retrospective nature of our study data analysis is susceptible to selection bias. Secondly, retrospective data acquisition may have impaired full access to all imaging records due to loss of follow-up or imaging performed prior to referral to our center. Thirdly, additional imaging studies may have been performed outside our surveillance program. Therefore, it is possible that our results over- or underestimate the number of surveillance CTUs required to detect one additional UTUC.

## 5. Conclusions

In our cohort, the incidence of UTUC during follow-up of NMIBC was only 1.2%. Only half of the patients diagnosed with UTUC were detected by routine upper-tract imaging alone while the other half was detected after the development of symptoms. UTUCs were detected exclusively in patients with initial HG disease, indicating that upper-tract surveillance might only be necessary in these patients. Risk-adapted follow-up strategies including grade, results of ultrasound, cytology and clinical signs should be evaluated in the future. The ideal approach would be to conduct a prospective study of predefined surveillance algorithms with and without CTU following a standardized protocol.

## Figures and Tables

**Table 1:** Patient characteristics.

**Number of patients**	315
Male	253 (80.3%)
Female	62 (19.7%)
**Age (y)**	66 (17)
**Overall follow-up (m)**	48 (55)
**Patients with recurrences**	43 (13.7%)
**Primary bladder tumor stage**	
Ta	207 (65.7%)
T1	98 (31.1%)
CIS	10 (3.2%)
**Highest bladder tumor stage during follow-up**	
Ta	201 (63.8%)
T1	103 (32.7%)
CIS	8 (2.5%)
≥T2a	3 (1%)
**Concomitant bladder carcinoma in situ (CIS)**	
Yes	42 (13.3%)
No	273 (86.7%)
**Primary bladder tumor grade (2004 Classification)**	
Low grade	149 (47.3%)
High grade	166 (52.7%)
**Highest bladder tumor grade during follow-up**	
Low grade	134 (42.5 %)
High grade	181 (57.5%)

Data presented as median (interquartile range) or number (percent).

**Table 2:** Results of diagnostic and pathology work-up of patients with UTUC during follow-up.

Age	Gender	Primary TNM	Highest TNM (Follow-Up)	Mode of UTUC Detection	Visible in US	Bladder Wash Cytology	Selective UT Cytology	UTUC Stage/Grade
68	F	Ta HG	Ta HG	CT	n/a	n/a	n/a	Ta LG
65	M	Ta HG	Ta HG	MRI	no	no malignant cells	no malignant cells	T1 LG
74	M	Ta HG	Ta HG	Macrohematuria	n/a	n/a	malignant cells	pT1 HG
80	F	Ta HG	T1 HG	Hydronephrosis	yes	malignant cells	malignant cells	pT3 HG

BC = Bladder Cancer; UTUC = Upper Urinary Tract Urothelial Carcinoma; MRI = Magnetic Resonance Imaging; CT = Computerized Tomography; US = Ultrasound; UT = Upper tract; LG = Low-grade; HG = High-grade.

## Data Availability

The data presented in this study are available on request from the corresponding author.

## Supplementary Materials

The following supporting information can be downloaded at: https://www.mdpi.com/article/10.3390/cancers14225586/s1, Supplemental Figure S1: Kaplan-Meier curve for UTUC free survival categorised by grade (HG and LG).

## References

[1] Bray F., Ferlay J., Soerjomataram I., Siegel R.L., Torre L.A., Jemal A. (2018). Global Cancer Statistics 2018: GLOBOCAN Estimates of Incidence and Mortality Worldwide for 36 Cancers in 185 Countries. CA. Cancer J. Clin..

[2] Babjuk M., Burger M., Capoun O., Cohen D., Compérat E.M., Dominguez Escrig J.L., Gontero P., Liedberg F., Masson-Lecomte A., Mostafid A.H. (2022). European Association of Urology Guidelines on Non–Muscle-Invasive Bladder Cancer (Ta, T1, and Carcinoma in Situ). Eur. Urol..

[3] Siegel R.L., Miller K.D., Jemal A. (2016). Cancer Statistics. CA Cancer J. Clin..

[4] Svatek R.S., Hollenbeck B.K., Holmäng S., Lee R., Kim S.P., Stenzl A., Lotan Y. (2014). The Economics of Bladder Cancer: Costs and Considerations of Caring for This Disease. Eur. Urol..

[5] Naser-Tavakolian A., Ghodoussipour S., Djaladat H. (2019). Upper Urinary Tract Recurrence Following Bladder Cancer Therapy. Curr. Opin. Urol..

[6] Millan-Rodriguez F., Chechile-Toniolo G., Salvador-Bayarri J., Huguet-Perez J., Vicente-Rodriguez J. (2000). Upper Urinary Tract Tumors after Primary Superficial Bladder Tumors: Prognostic Factors and Risk Groups. J. Urol..

[7] Rabbani F., Perrotti M., Russo P., Herr H.W. (2001). Upper-Tract Tumors after an Initial Diagnosis of Bladder Cancer: Argument for Long-Term Surveillance. J. Clin. Oncol..

[8] Golabesk T., Palou J., Rodriguez O., Parada R., Skrobot S., Peña J.A., Villavicencio H. (2017). Long-Term Bladder and Upper Urinary Tract Follow-up Recurrence and Progression Rates of G1-2 Non-Muscle-Invasive Urothelial Carcinoma of the Bladder. Urology.

[9] Herr H.W. (1998). Extravesical Tumor Relapse in Patients with Superficial Bladder Tumors. J. Clin. Oncol..

[10] Kirkali Z., Tuzel E. (2003). Transitional Cell Carcinoma of the Ureter and Renal Pelvis. Crit. Rev. Oncol. Hematol..

[11] Power N.E., Izawa J. (2016). Comparison of Guidelines on NonMuscle Invasive Bladder Cancer (EAU, CUA, AUA, NCCN, NICE). Bladder Cancer.

[12] Jinzaki M., Matsumoto K., Kikuchi E., Sato K., Horiguchi Y., Nishiwaki Y., Silverman S.G. (2011). Comparison of CT Urography and Excretory Urography in the Detection and Localization of Urothelial Carcinoma of the Upper Urinary Tract. Am. J. Roentgenol..

[13] Wang L.J., Wong Y.C., Huang C.C., Wu C.H., Hung S.C., Chen H.W. (2010). Multidetector Computerized Tomography Urography Is More Accurate Than Excretory Urography for Diagnosing Transitional Cell Carcinoma of the Upper Urinary Tract in Adults With Hematuria. J. Urol..

[14] Silverman S.G., Leyendecker J.R., Amis E.S. (2009). What Is the Current Role of CT Urography and MR Urography in the Evaluation of the Urinary Tract?. Radiology.

[15] Smith-Bindman R., Lipson J., Marcus R., Kim K.P., Mahesh M., Gould R., Berrington De González A., Miglioretti D.L. (2009). Radiation Dose Associated With Common Computed Tomography Examinations and the Associated Lifetime Attributable Risk of Cancer. Arch. Intern. Med..

[16] O’Sullivan J.W., Muntinga T., Grigg S., Ioannidis J.P.A. (2018). Prevalence and Outcomes of Incidental Imaging Findings: Umbrella Review. BMJ.

[17] Eble J.N., Sauter G., Epstein J.I., Sesterhenn I.A. (2004). Pathology and Genetics of Tumours of the Urinary System and Male Genital Organs WHO OMS.

[18] Mostofi F.K., Davis C.J., Sesterhenn I.A., Sobin L.H. (1973). Histological Typing of Urinary Bladder Tumours.

[19] Molen A.J., Cowan N.C., Mueller-Lisse U.G., Nolte-Ernsting C.C.A., Takahashi S., Cohan R.H. (2008). CT Urography: Definition, Indications and Techniques. A Guideline for Clinical Practice. Eur. Radiol..

[20] Oldbring J., Glifberg I., Mikulowski P., Hellsten S. (1989). Carcinoma of the Renal Pelvis and Ureter Following Bladder Carcinoma: Frequency, Risk Factors and Clinicopathological Findings. J. Urol..

[21] Holmang S., Hedelin H., Anderstrom C., Holmberg E., Johansson S.L., Holmäng S., Hedelin H., Anderström C., Holmberg E., Johansson S.L. (1998). Long-Term Followup of a Bladder Carcinoma Cohort: Routine Followup Urography Is Not Necessary. J. Urol..

[22] Nishiyama N., Hotta H., Takahashi A., Yanase M., Itoh N., Tachiki H., Miyao N., Matsukawa M., Kunishima Y., Taguchi K. (2018). Upper Tract Urothelial Carcinoma Following Intravesical Bacillus Calmette-Guérin Therapy for Nonmuscle-Invasive Bladder Cancer: Results from a Multi-Institutional Retrospective Study. Urol. Oncol. Semin. Orig. Investig..

[23] Hurle R., Losa A., Manzetti A., Lembo A. (1999). Upper Urinary Tract Tumors Developing after Treatment of Superficial Bladder Cancer: 7-Year Follow-up of 591 Consecutive Patients. Urology.

[24] Canales B.K., Anderson J.K., Premoli J., Slaton J.W. (2006). Risk Factors for Upper Tract Recurrence in Patients Undergoing Long-Term Surveillance for Stage Ta Bladder Cancer. J. Urol..

[25] Lin N., Wu Y.-P., Lin Y.-Z., Tao X., Chen S.-H., Ke Z.-B., Wei Y., Zheng Q.-S., Xue X.-Y., Xu N. (2018). Risk Factors for Upper Tract Urothelial Recurrence Following Local Excision of Bladder Cancer. Cancer Med..

[26] Mangrud O.M., Waalen R., Gudlaugsson E., Dalen I., Tasdemir I., Janssen E.A.M., Baak J.P.A. (2014). Reproducibility and Prognostic Value of WHO1973 and WHO2004 Grading Systems in TaT1 Urothelial Carcinoma of the Urinary Bladder. PLoS ONE.

[27] Sternberg I.A., Paz G., Chen L.Y., Keren Paz G.E., Chen L.Y., Herr H.W., Donat S.M., Bochner B.H., Dalbagni G. (2013). Upper Tract Imaging Surveillance Is Not Effective in Diagnosing Upper Tract Recurrence in Patients Followed for Nonmuscle Invasive Bladder Cancer. J. Urol..

[28] Ayyathurai R., Soloway M.S. (2011). Monitoring of the Upper Urinary Tract in Patients with Bladder Cancer. Indian J. Urol..

[29] Herr H.W., Donat S.M. (1999). Prostatic Tumor Relapse in Patients with Superficial Bladder Tumors: 15-Year Outcome. Juro.

[30] Yousem D.M., Gatewood O.M.B., Goldman S.M., Marshall F.F. (1988). Synchronous and Metachronous Transitional Cell Carcinoma of the Urinary Tract: Prevalence, Incidence, and Radiographic Detection. Radiology.

