# Supplementary material for: Is Regular Radiographic Upper Urinary Tract Imaging for Surveillance of Non-Muscle Invasive Bladder Cancer Justified?
Source: Cancers (Basel). 2022 Nov 14;14(22):5586. doi: 10.3390/cancers14225586 (PMC9688179; doi:10.3390/cancers14225586)
Supplement: Supplementary file 1 [file cancers-14-05586-s001.zip › cancers-1979341-supplementary.pdf]

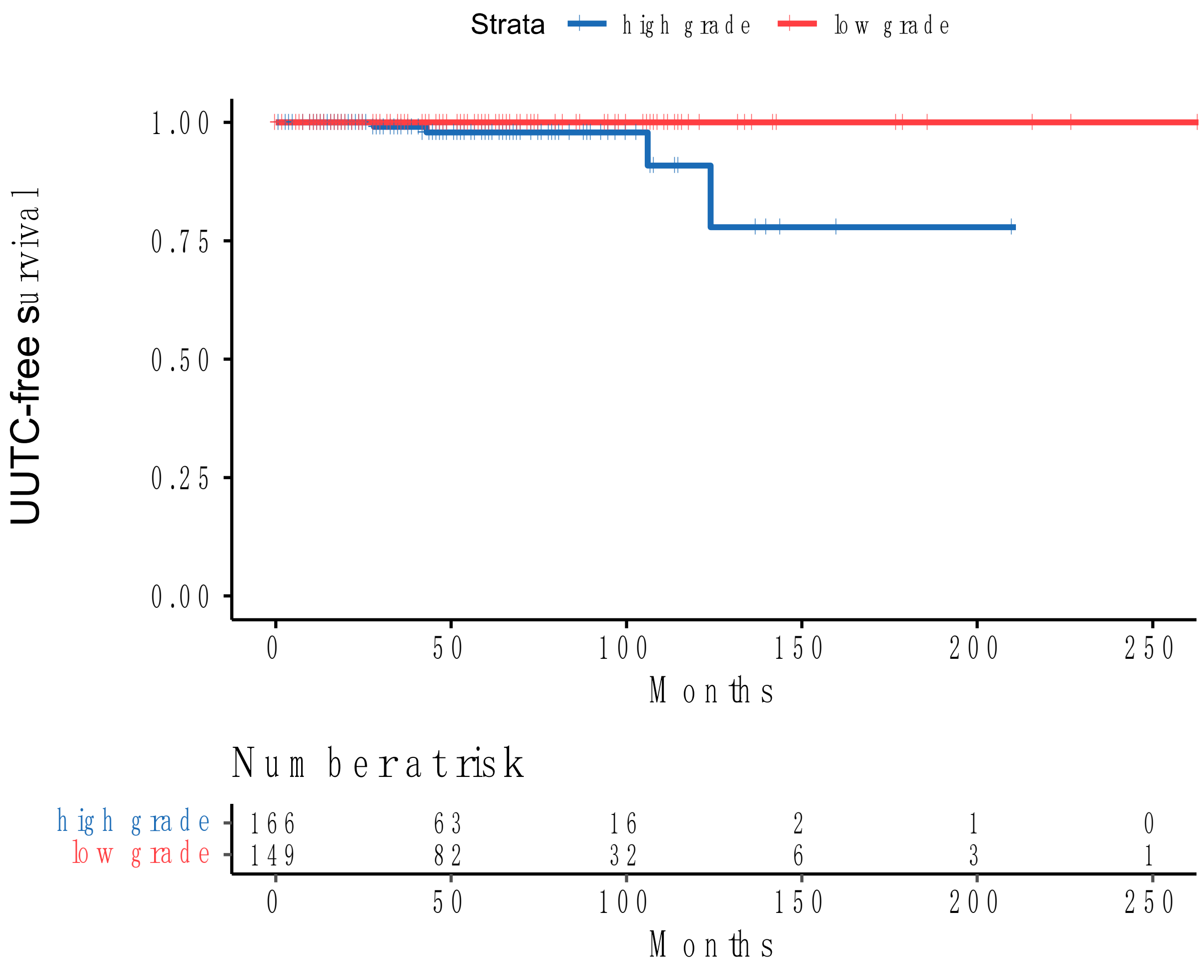

Supplemental Figure S1. Kaplan-Meier curve for UTUC free survival categorised by grade (HG and LG). The red and blue band bordering the curve marks the 95% confidence interval; censored subjects are indicated as tick marks.
